# Recombinant Erythropoietin Provides Protection against Renal Fibrosis in Adenine-Induced Chronic Kidney Disease

**DOI:** 10.1155/2020/8937657

**Published:** 2020-02-27

**Authors:** Estefanía Vázquez-Méndez, Yanet Gutiérrez-Mercado, Edgar Mendieta-Condado, Francisco Javier Gálvez-Gastélum, Hugo Esquivel-Solís, Yadira Sánchez-Toscano, Claudia Morales-Martínez, Alejandro A. Canales-Aguirre, Ana Laura Márquez-Aguirre

**Affiliations:** ^1^Unidad de Biotecnología Médica y Farmacéutica, Centro de Investigación y Asistencia en Tecnología y Diseño del Estado de Jalisco, A.C, Guadalajara, Jalisco, Mexico; ^2^Doctorado en Farmacología, Centro Universitario de Ciencias de la Salud, Universidad de Guadalajara, Jalisco, Mexico; ^3^Departamento de Biología Molecular y Validación de Técnicas, Instituto de Diagnóstico y Referencia Epidemiológicos-SSA, Mexico; ^4^Departamento de Microbiología y Patología, Centro Universitario de Ciencias de la Salud, Universidad de Guadalajara, Jalisco, Mexico; ^5^Escuela de Ciencias de la Salud, Universidad Guadalajara LAMAR, Campus Vallarta, Guadalajara, Mexico

## Abstract

Chronic kidney disease (CKD) causes anemia by renal damage. In CKD, the kidney is submitted to hypoxia, persistent inflammation, leading to fibrosis and permanent loss of renal function. Human recombinant erythropoietin (rEPO) has been widely used to treat CKD-associated anemia and is known to possess organ-protective properties that are independent from its well-established hematopoietic effects. Nonhematopoietic effects of EPO are mediated by an alternative receptor that is proposed to consist of a heterocomplex between the erythropoietin receptor (EPOR) and the beta common receptor (*β*cR). The present study explored the effects of rEPO to prevent renal fibrosis in adenine-induced chronic kidney disease (Ad-CKD) and their association with the expression of the heterodimer EPOR/*β*cR. Male Wistar rats were randomized to control group (CTL), adenine-fed rats (Ad-CKD), and Ad-CKD with treatment of rEPO (1050 IU/kg, once weekly for 4 weeks). Ad-CKD rats exhibited anemia, uremia, decreased renal function, increased infiltration of inflammatory cells, tubular atrophy, and fibrosis. rEPO treatment not only corrected anemia but reduced uremia and partially improved renal function as well. In addition, we observed that rEPO diminishes tubular injury, prevents fibrosis deposition, and induces the EPOR/*β*cR heteroreceptor. The findings may explain the extrahematopoietic effects of rEPO in CKD and provide new strategies for the treatment of renal fibrosis in CKD.

## 1. Introduction

Chronic kidney disease (CKD) is currently defined as a progressive and irreversible decline in glomerular filtration rate (GFR) below 60 mL/min per 1.73 m^2^ of body surface. Irrespective of the underlying cause, the series of events that lead to end-stage kidney disease, and thus the need for replacement therapy, are an initial injury by hypoxia or toxins, followed by chronic inflammation, oxidative stress, vascular remodeling, and finally tubular and glomerular fibrosis [1]. CKD is a leading cause of morbidity and mortality worldwide, as hypertension and diabetes are the most common causes. It is estimated that 10-15% of the population in industrialized countries develop some degree of CKD [2]. The burden of this disease and its complications pose a growing problem to society as the incidence of CKD increases at an annual rate of 8% and consumes up to 2% of the global health expenditure [3]. In addition to addressing the causes of CKD, a higher understanding of the pathophysiological mechanisms that underlie progression of the disease needs to be achieved.

Human recombinant erythropoietin (rEPO) has been widely used to treat anemia associated with CKD. Cells of erythroid lineage express the erythropoietin receptor (EPOR) to promote differentiation, survival, and expansion of cell population in response to hypoxia. However, in addition to its well-known role in erythropoiesis, a diverse array of cells have been identified that produce EPO and/or express the EPOR, including endothelial cells, smooth muscle cells, tubular kidney cells, and cells of the central nervous system [4–6].

Recent studies have shown that supratherapeutic doses of rEPO can exert cytoprotective effects in some tissues, kidney tissue included, by activating the heterodimer formed by the interleucine-3 *β* common receptor (*β*cR) and the EPOR [7–9]. There is evidence that EPO derivatives can ameliorate kidney cell damage by upregulating nitric oxide production and strengthening antiapoptotic processes [7, 10, 11] as well as by downregulating the transdifferentiation of epithelial and endothelial cells into activated myofibroblasts that contribute to excessive extracellular matrix formation and ultimately tubulointersticial fibrosis [12, 13].

Renal fibrosis is a major contributor to the development of CKD, and given the significance of EPOR/*β*cR heterodimer in EPO-conferred nonhematopoietic protection, in the present study, we aimed to investigate how the process of tubulointersticial fibrosis can be influenced by the coexpression of the EPOR/*β*cR heterodimer and the administration of rEPO in Ad-CKD model.

## 2. Materials and Methods

### 2.1. Animals and Experimental Groups

The present study was approved by the Institutional Animal Care Committee and complied with the national normativity (NOM-062-ZOO-1999) (approval number 2016-019). Male Wistar rats weighing 250-300 g were purchased from Envigo (Mexico City, Mexico). They were housed in a climate-controlled and light-regulated facility with 12/12 h day-night cycles and were fed regular rat chow and water ad libitum. After an acclimatization period of one week, 24 rats were randomly divided into four study groups: control untreated rats (CTL), adenine-induced chronic kidney disease (Ad-CKD), and Ad-CKD+rEPO. Administration of adenine, either in food or by oral gavage, has been shown to result in reproducible renal dysfunction in previous studies [14–19]. In this study, CKD was induced by oral gavage of adenine (100 mg/kg/day; Sigma-Aldrich #A8626) for a period of 28 days. Rats of the control group (CTL) were treated daily with vehicle (water). The rEPO treatment (Exetin®, PiSA Laboratories) was performed simultaneously with the administration of adenine. A dose of 1050 IU/kg of rEPO was administered subcutaneously once a week (four administrations in total). Previous dose selection test and rEPO kinetics in serum were performed and are shown as supplementary material ([Supplementary-material supplementary-material-1]).

### 2.2. Sample Collection

During the treatment period, rats were weighed weekly; one day before the last day of treatment, rats were placed individually in metabolic cages to collect 24-hour urine. At the end of the study, rats were anesthetized with xylazine (5 mg/kg) and ketamine (75 mg/kg) intraperitoneally and blood samples were collected by cardiac puncture and placed into plain tubes with and without anticoagulant. Blood samples were used for biochemical and complete blood count. Both kidneys were excised, blotted on filter paper, and weighed. Coronal sections of the right kidneys were fixed in 4% neutral-buffered paraformaldehyde for subsequent histopathology, and the rest of the kidneys were kept frozen at −80°C until use.

### 2.3. Hematological and Biochemical Parameters

#### 2.3.1. Hematology

Blood with anticoagulant was recollected for erythrocyte count (EC), hemoglobin (Hb) concentration, hematocrit (packed cell volume, PCV), and reticulocytes, using automated Coulter method with *SYSMEX kx-21*N® equipment.

#### 2.3.2. Biochemistry

24 h urine samples were used to determine 24 h urinary volumes (uV) and urine creatinine (UCr) levels. Serum creatinine (SCr) and urea were also determined using a *SYSMEX kx-21*N® dry chemistry analyzer. GFR was calculated with the following equation: GFR = (UCr∗uV)/(SCr) [20].

### 2.4. Renal Histology

Fixed kidneys were cryoprotected (30% sucrose in PBS, pH 7.4) overnight at 4°C, embedded in Tissue Tek (O.C.T. Compound Sakura) and snap frozen at -25°C. Frozen tissue (10 *μ*m) was sectioned with a cryostat and then stained with hematoxylin and eosin (H&E) or Masson's trichrome stain. The microscopic scoring of the kidney sections was carried out in a blinded fashion by a pathologist who was unaware of the treatment groups; 20 visual fields were analyzed per tissue. Each section was assigned a score to represent the approximate extent of renal tubule damage on a scale of 0–4: 0, no damage; 1, a few focal damaged areas (<25%); 2, damaged area is about one-half of the section area (25-50%); 3, the damaged area occupied about two-thirds of the section area (50-75%); and 4, nearly the entire area was damaged (>75%). An additional score measuring the extent of fibrosis was given on a scale of 0–4: 0, no fibrosis; 1, mild fibrosis; 2, fibrosis observed in about one-half of the section; 3, fibrosis observed in about two-thirds of the section; and 4, nearly the entire area was fibrosed [16, 21]. The inflammatory infiltrate was assessed as a scale: 1, no infiltrate; 2 little infiltrate (10% of field); 3, moderate infiltrate (10–50% of field); and 4, extensive infiltrate (50% of field and usually 50% of the field). The final score was then calculated as a mean of scale [22].

### 2.5. RNA Extraction and Real-Time PCR

Total RNA was isolated from the renal cortex using a TRIzol reagent (Invitrogen, Life Technologies, CA, USA), and retrotranscription was performed using 2 mg of total RNA and M-MLV reverse transcriptase (Invitrogen, Life Technologies, CA, USA). Two microliters of the cDNA reaction mixture was subjected to real-time PCR under the following conditions: preincubation 10 min/95°C and 45 cycles of 10 s/95°C, 10 s/60°C, and 10 s/72°C using TaqMan probes (Applied Biosystems, Life Technologies, CA, USA) to amplify collagen type 1*α* (Col-1*α*), transforming growth factor beta (TGF-*β*), and 18S rRNA housekeeping gene as the normalization control. Relative quantification of gene expression was performed with the comparative CT method (*ΔΔ*CT method). The expression profile for each gene was reported as fold change = 2‐ΔΔCT plus standard deviation calculated as *σ* = √(*σ*(18S gene)^2^ + *σ*(Target gene)^2^), according to the Applied Biosystems User Bulletin #2.

### 2.6. Western Blot and Immunoprecipitation

Kidney cortical tissue was homogenized with RIPA buffer (Sigma-Aldrich, R0278) on ice; total protein was quantified by BCA method (Pierce, #23225). For western blot, prepared samples were loaded on a 10% Tris-glycine polyacrylamide gel. Precision Plus Protein All Blue Standards (Bio-Rad) marker was included in at least two lanes on all gels. After separation by electrophoresis, proteins were transferred to a polyvinylidene difluoride (PVDF) membrane. Blots were blocked in 3% dry milk blocking buffer (prepared in Tris-buffered saline with 0.1% Tween 20) and were incubated overnight at 4°C with EPO-R antibody 1 : 200 (Santa Cruz Biotechnology, sc-82593) and IL-3R*β* (*β*cR) antibody 1 : 200 (Santa Cruz Biotechnology, sc-677); *β*-tubulin (1 : 200) was used as control (Santa Cruz Biotechnology, sc-23949). Subsequently, the membrane was washed in TBS-T 0.5% 5 times and incubated with HRP IgG goat anti-rabbit secondary antibody 1 : 10000 (Vector Laboratories PI 1000) or HRP horse anti-mouse IgG antibody 1 : 5000 (Vector Laboratories PI 2000). Images obtained from blots were quantified using ImageLab software version 6.0.1 (Bio-Rad, CA); statistical analysis was performed with Sigma Stat version 1.1. All protein expression was normalized using *β*-tubulin control and expressed as relative units (R.U.). For immunoprecipitation, cellular lysates in RIPA buffer were incubated with anti-IL-3R*β* (*β*cR) (Santa Cruz; sc-398246) overnight at 4°C in gentle shaking (1 *μ*g IgG per 100 *μ*g in 1 mL cell lysate) then immunoprecipitated (IP) with 20 *μ*L of A/G-plus agarose (Sc-2003; Santa Cruz) per mL and immunoblotted with anti-EPOR (Santa Cruz; sc-365662).

### 2.7. Immunofluorescence

Primary antibodies were applied onto prefixed frozen tissue sections as follows: aquaporin 1 : 1.250 (Santa Cruz Biotechnology, sc-9878), *α*-smooth muscle actin 1 : 1000 (Santa Cruz Biotechnology, sc-53016), collagen 1*α* 1 : 1000 (Thermo Fisher, PA1-27397), EPOR 1 : 500 (Santa Cruz Biotechnology, sc-82593), and IL-3R*β* (*β*cR) (Santa Cruz Biotechnology, sc-677). Images were obtained with confocal microscopy (Leica TCS SPE DM5500, software LAS X) at a 40x magnification, 3 cortical fields analyzed per sample.

### 2.8. Statistical Analyses

Statistical analysis was carried out using GraphPad Prism 6.0 (GraphPad Software, San Diego, CA, USA). Values are presented as means ± standard deviations unless otherwise specified. To compare the effect of treatment between groups, a one-way ANOVA was performed with a *post hoc* Tukey test. *P* values of <0.05 were considered statistically significant.

## 3. Results

### 3.1. Erythropoietin Corrects Anemia and Improves Kidney Function

Ad-CKD rats displayed expected metabolic changes with the disease state; the clinical presentation of kidney disease manifested as a hyperfiltration state, similar to the initiating phases of diabetic kidney disease [23]. As shown in Table 1, adenine feeding caused a significant decrease in body weight (82% decrease vs. control) and food intake (57% decrease vs. control) and a significant increase in water intake (244% increase vs. control) and urine output (388% increase vs. control) (*P* < 0.05). These changes were significantly but not completely antagonized by rEPO treatment. Since adenine intoxication induces chronic kidney damage and depletes the number of functional EPO-producing fibroblasts, hemoglobin, hematocrit, and erythrocyte indexes descend as a consequence. Adenine feeding caused significant decreases in EC, Hb, and reticulocytes (*P* < 0.05). These changes were completely corrected by rEPO treatment: Hb 13.3 vs. 17.8 g/dL, EC 8.8 vs. 6.7 × 10^6^/*μ*L, Htc 41.3 vs. 58.2%; in all cases, *P* < 0.05 (Ad-CKD vs. Ad-CKD+rEPO). Adenine intoxication significantly increased kidney weight and size and increased the concentrations of serum urea and creatinine. rEPO significantly ameliorated these changes in the adenine-treated rats, where serum creatinine decreased from 2.19 to 0.93 mg/dL, urea descended from 196.6 to 78.2 mg/dL (*P* < 0.05 in both cases). Finally, GFR showed a significant decrease in the Ad-CKD group vs. control from 0.73 to 0.24 mg/min/g (*P* < 0.05), whereas Ad-CKD+rEPO did not.

### 3.2. Erythropoietin Attenuates Tubular Damage and Prevents Renal Fibrosis

**Figure 1:**
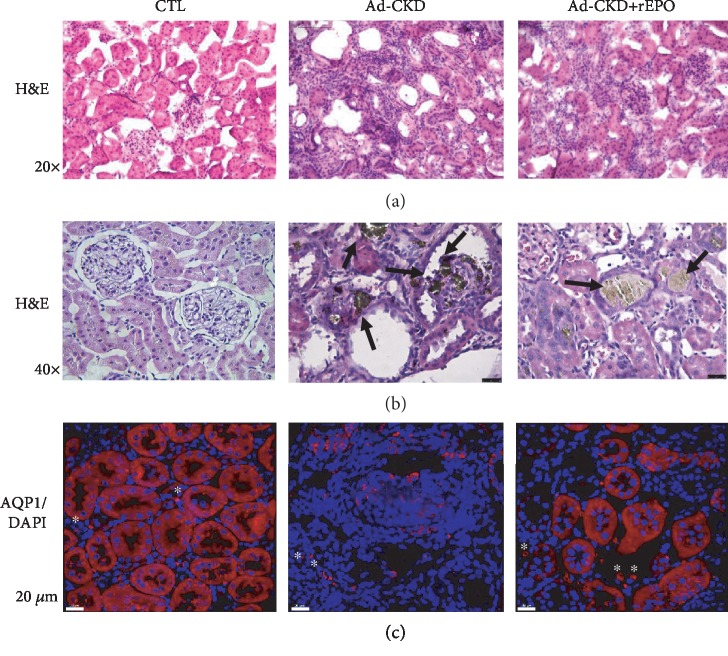
rEPO reduces inflammatory infiltrate and tubular atrophy in Ad-CKD rats. Micrographs of kidney sections. (a, b) Microphotographs stained with hematoxylin-eosin observed under a light microscope (H&E, 20x and 40x). (b) Control group (CTL), no histological changes were observed; adenine-fed rats (Ad-CKD) showed remarkable histological changes characterized by increased inflammatory infiltrate (purple dots), interstitial expansion, tubular dilatation, and atrophy as well characteristic tubular casts (black arrows). (c) AQP1/DAPI immunostain. The Ad-CKD group exhibited less AQP1 expression (tubular atrophy), whereas rEPO treatment maintains the expression of AQP1 (3 cortical fields per animal were analyzed) (40x). ^∗^Red blood cells. Scale bar is 20 *μ*m.

Adenine-induced nephropathy is well known to generate progressive and reproducible renal dysfunction in animal models initiated by the deposition of 2,8-dihydroxyadenine crystals in the renal interstitial space, promoting migration of immune cells and thus initiating a cascade of inflammatory events that ultimately result in chronic hypoxia, tubular atrophy, uremia, and fibrosis [14, 17]. The adenine-treated group exhibited tubular distention with loss of brush border of proximal tubules, dilatation of a large number of tubules, inflammatory cell infiltration of the interstitium (about 75% of total fields), and focal tubular atrophy (damage score of 4). The rats given adenine plus rEPO on the other hand displayed improvement in the histological appearance when compared with the adenine-treated group. There were less dilatation of the tubules, less interstitial inflammatory cell infiltration, and less tubular atrophy (damage score of 3) (Figure 1(a)). Characteristic tubular casts, presumably containing 2,8-dihydroxyadenine, were also observed in both adenine-treated groups (Figure 1(b)). In order to corroborate the improvement in the histological changes, we performed immunofluorescence (IF) staining to aquaporin (AQP1), as a main marker of proximal tubular cells. The Ad-CKD group exhibited less AQP1 expression (tubular atrophy), whereas rEPO treatment maintains the expression of AQP1 (Figure 1(c)).

**Figure 2:**
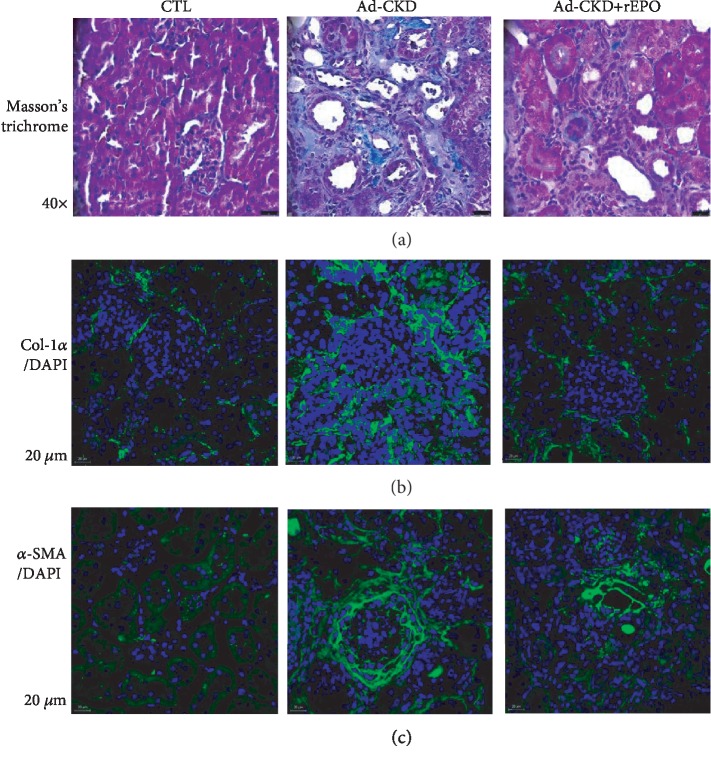
rEPO prevents renal fibrosis and reduces *α*-SMA and Col-1 expression in Ad-CKD rats. (a) Masson's trichrome stain (40x), micrographs of kidney sections. Control group (CTL), no histological changes were observed; adenine-fed rats (Ad-CKD) showed remarkable interstitial fibrosis (blue). Ad-CKD rats treated with rEPO (Ad-CKD+rEPO) displayed less fibrosis area. (b) Collagen 1*α*/DAPI staining. Dot-like staining can be seen in the control group; however, the Ad-CKD group displays continuous collagen deposition in the interstitial space, whereas the Ad-CKD+rEPO group collagen deposition only outlines tubular structure. (c) *α*-SMA/DAPI staining. No interstitial staining can be seen in the control group but the Ad-CKD group displays net-like staining around tubules and glomeruli (3 cortical fields per animal were analyzed) (40x). Scale bar is 20 *μ*m.

On the other hand, tissues were also stained with Masson's trichrome to examine fibrosis. Masson's trichrome stains type I collagen as well as variety of extracellular matrix elements. There was little blue staining in sections from control rats, except for the expected perivascular staining around the intrarenal arteries. In contrast, there was considerable positive staining in the interstitial space of adenine-treated rats. rEPO treatment ameliorated these changes (damage score of 3) (Figure 2(b)).  Table 2 depicts a qualitative score evaluation of findings regarding tubular atrophy, inflammatory infiltrate, and fibrosis.

We also performed immunofluorescence (IF) to *α*-SMA and Col-1*α*, specific markers of tubulointerstitial fibrosis. Collagen 1 surrounds all visible tubules and it occupies all the interstitial space in Ad-CKD; rEPO treatment shows a diminished intensity of collagen staining compared to the Ad-CKD group, even though it displays the same pattern of deposition (Figure 2(b)). *α*-SMA acquires a lineal structure that surrounds glomeruli in a continuous form in the Ad-CKD group; however, in the Ad-CKD+rEPO group, *α*-SMA reactivity is limited to surrounding vascular structures and does not reach the glomeruli (Figure 2(c)).

**Figure 3:**
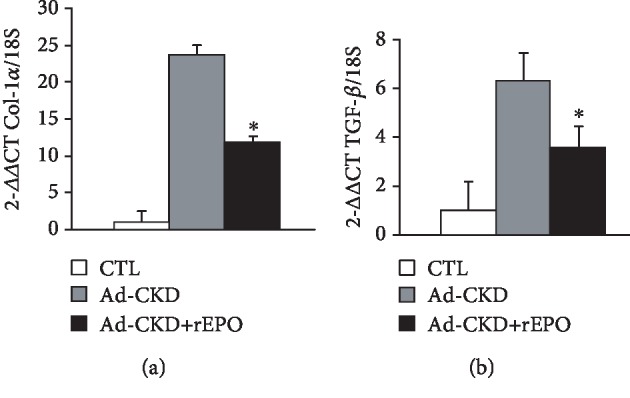
mRNA Col-1*α* and TGF-*β* expression in the kidney. Gene expression was assessed by real-time QPCR, revealing significantly increased expression of collagen 1 and TGF-*β* in the adenine-fed rats compared with control. (a) rEPO treatment reduces significantly (^∗^*P* < 0.05) the expression of (a) Col-1 and (b) TGF-*β* in the kidney cortex compared with Ad-CKD.

Finally, mRNA expression of fibrosis markers Col-1*α* and TGF-*β* was determined by QPCR. As shown in Figure 3, we observed significant upregulation of Col-1*α* (Figure 3(a)) and TGF-*β* (Figure 3(b)) in Ad-CKD. However, mRNA expression levels for Col-1*α* and TGF-*β* were significantly reduced in Ad-CKD+rEPO, compared with Ad-CKD. Masson's staining, immunofluorescence, and QPCR findings confirm that fibrosis is attenuated by rEPO treatment.

### 3.3. rEPO Induced Coexpression of the Tissue-Protective Receptor in Ad-CKD Rats

**Figure 4:**
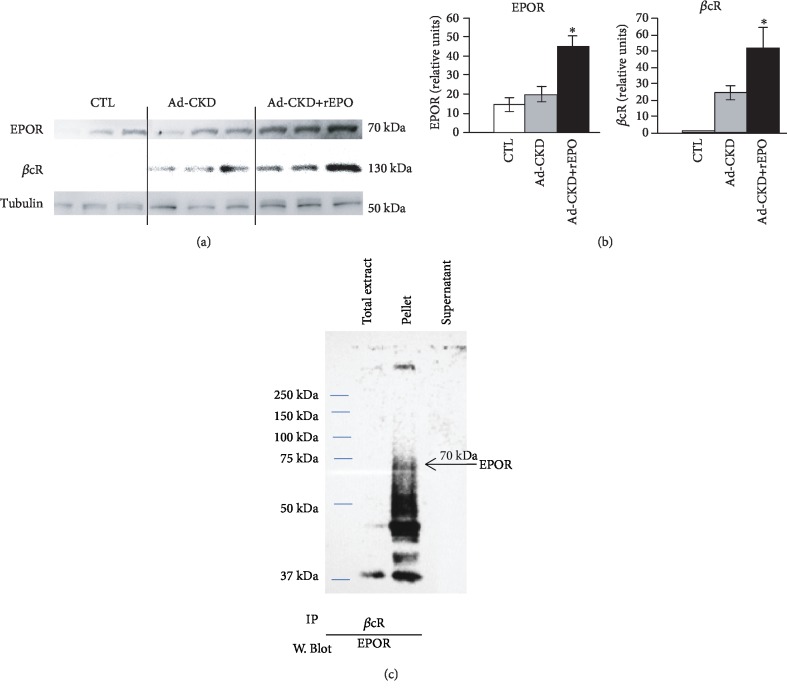
rEPO increased EPOR and *β*cR protein expression and induced coexpression in Ad-CKD rats. (a) Representative western blotting image of EPOR and *β*cR and *β*-tubulin. (b) Relative expression of EPOR and *β*cR. Significant difference in the Ad-CKD+rEPO group (^∗^*P* < 0.05). (c) Immunoprecipitation, cellular lysates were incubated with anti-*β*cR Ab overnight at 4°C in gentle shaking (1 *μ*g IgG per 100 *μ*g in 1 mL cell lysate) then immunoprecipitated (IP) with 20 *μ*L of A/G-plus agarose per mL and immunoblotted with anti-EPOR.

Based on immunoprecipitation studies, Brines and coworkers proposed that the nonhematopoietic effects of EPO were mediated by a heterodimer of EPOR and the beta common (*β*c) receptor which was referred to as the “Innate Repair Receptor” (IRR) [8]. In our study, we performed western blot for each receptor and a coimmunoprecipitation (Figure 4). On the one hand, we performed a quantification of EPOR and *β*cR in homogenized cortical kidney tissue by western blot. We found that EPOR protein was expressed in all groups, but it is increased significantly by rEPO treatment. On the other hand, *β*cR expression was not observed in the control group; this protein was only expressed in the Ad-CKD group and more significantly in the Ad-CKD+rEPO group (Figures 4(a) and 4(b)). Western blot analysis of proteins immunoprecipitated from kidneys expressing both receptors (Ad-CKD+rEPO group) with antibody against *β*cR and blotted with antibody against EPOR was performed. These data show that the *β*cR chain associates with the EPOR in Ad-CKD+rEPO (Figure 4(c)).

**Figure 5:**
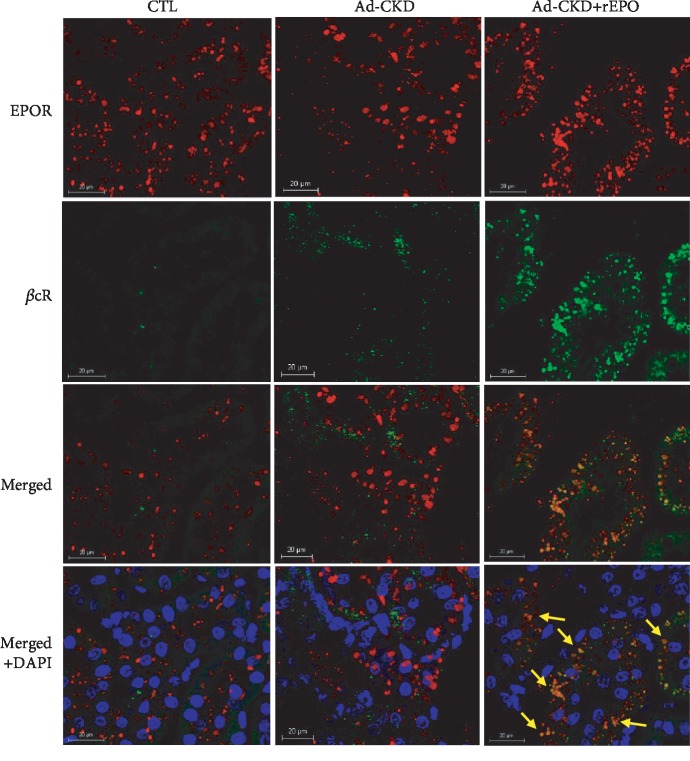
rEPO induces colocalization of EPOR and *β*cR in Ad-CKD rats. Immunofluorescence staining micro images for EPOR (first line), *β*cR (second line), merged images of EPOR/*β*cR (third line), and DAPI (fourth line) (40x). Scale bar is 20 *μ*m. The control group shows EPOR reactivity, no *β*cR, and thus no coexpression. The Ad-CKD group displays EPOR some *β*cR and no coexpression. The Ad-CKD+rEPO group shows increased expression of EPOR and *β*cR and coexpression of the heteroreceptor as depicted by orange dots (yellow arrows).

Once we asserted the expression of IRR in kidney tissue, we proceeded to evaluate colocalization through immunofluorescence staining (Figure 5). In the CTL and Ad-CKD groups, EPOR expression can be seen around most visible tubules; however, as previously observed in western blot, rEPO treatment induces an increased expression of EPOR in renal cells in Ad-CKD rats (Figure 5, line 1). Moreover, *β*cR only displays some unspecific staining (likely only in erythrocytes) in the interstice in the CTL group, and in Ad-CKD rats, this receptor shows scant diffuse expression; conversely, rEPO treatment induces and increases expression in renal cells (Figure 5, line 2). Although the Ad-CKD group displays some EPOR and *β*cR expression, when merging images, coexpression of both receptors cannot be seen (Figure 5, lines 3 and 4, in the middle) (Supplementary file, [Supplementary-material supplementary-material-1]). Interestingly, in the Ad-CKD+rEPO group, both EPOR and *β*cR are evidently expressed in renal cells, and when merging images, coexpression of both receptors is evident, represented by yellow-orange dots (Figure 5, lines 3 and 4, on the right side) (Supplementary file, [Supplementary-material supplementary-material-1]). This finding correlates with the data from the western blot and immunoprecipitation and confirms that IRR was expressed and increased in Ad-CKD+rEPO.

## 4. Discussion

Adenine-induced CKD was manifested in rats by metabolic and hematologic changes and kidney function decline that was ameliorated by rEPO treatment. Anemia, evident at the final time point of the study, was successfully corrected by rEPO treatment; furthermore, the 1050 IU/kg dose used did not induce polycythemia or other evident thrombotic adverse events in any of the study groups. Adequate response to rEPO treatment is relevant since low hemoglobin levels can perpetuate hypoxia and negatively modulate cellular signaling mechanisms associated with the development of progenitor stem cells, cellular integrity, and angiogenesis [24]. Several preclinical as well as clinical studies have shown that rEPO can prevent renal function deterioration in acute kidney injury [25]; however, maintenance of kidney function once chronic disease has been implemented (as evidenced by the formation of tissue fibrosis) is not easily achieved. In our study, creatinine clearance, urea, and GFR were significantly improved by rEPO treatment, resembling levels in control groups. The decline in GFR in kidney disease can be associated with the accumulation of uremic retention solutes that have detrimental effects in cellular metabolic processes, inflammation, and cell survival [26]. Uremic toxins can elicit direct toxicity on epithelial intestinal cells, adipocytes, and vascular endothelial wall cells and indirect toxicity by eliciting protein carbamylation of proteins [27]. Studies performed on cellular cultures as well as animal models have shown that carbamylated albumin can induce a fibrogenic state by promoting expression of inflammatory cytokines (through NF-*κ*b) and growth factors, such as transforming growth factor *β* (TGF-*β*) and epidermal growth factor (EGF) and endothelin-1 by tubular cells [28]. rEPO has been shown to decrease levels of creatinine and urea in animal models of acute kidney disease with hypoxia or sepsis. Knocking down the *β*cR gene in mice hindered the protective effect conferred by rEPO [7, 29]. In our study, rEPO significantly reduced urea and serum creatinine in Ad-CKD rats and considerably improved GFR while inflammatory cell infiltrate was reduced. This could have been influenced by the lower uremic toxins in serum.

The chain of events that lead to fibrosis in most forms of chronic kidney disease are inflammatory cell infiltration, mesangial and fibroblast infiltration, tubular epithelial to mesenchymal transition, and extracellular matrix expansion [30]. Infiltration of inflammatory cells into the interstice initiates by recruitment from the vascular space of neutrophils, macrophages, and lymphocytes. Studies evaluating the effect of rEPO or an EPO derivative ARA290 have shown that rEPO inhibits macrophage activation while promoting phagocytic activity through activation of the EPOR/*β*cR heterodimer in experimental models of atherosclerosis and autoimmune neuritis [31, 32].

As insult persisted by daily administration of adenine, inflammatory cells became permanent residents in kidney tissue (Figure 1) and brought about an environment of constant formation of oxygen radicals in which tubular cells attempt to survive by activating proliferation, differentiation and stress-induced senescence, EMT, and finally apoptosis. Supratherapeutic doses of rEPO have proven to reduce tubular atrophy, glomerular sclerosis, and fibrosis by downregulating the expression of profibrogenic proteins TGF-*β*, Smad 2/3 in a unilateral ureteral obstruction model of kidney disease [33]. Indeed, the protective effects of a synthetic form of rEPO on a mice model of diabetic nephropathy confirm that tubulointersticial fibrosis, triggered by Snail/Smad 3/TGF-*β* upregulation, leads to EMT as a sequence of the following events: (1) loss of epithelial or endothelial characteristics, (2) *α*-SMA expression and reorganization of actin fibers, (3) alterations of the basement membrane, and (4) cell migration and replacement of normal resident cells of the interstitium [15, 33]. Chronic injurious stimuli which originated from persistent inflammation result in an excess deposition of extracellular matrix components.

The tissue-protective receptor is a heterocomplex composed of EPOR and the *β*-common receptor (*β*cR or CD131) that exhibits a lower affinity for EPO. In normal circumstances, in order to maintain stable levels of red blood cells, plasma levels of EPO remain constant in the 1-10 pmol/L range; however, a much higher concentration of EPO of about 1-2 nmol/L is required in nonhematopoietic cells to initiate cell signaling mechanisms that confer tissue protective effects. In contrast with the autocrine function in hematopoietic cells that require consistent activation of the EPOR dimer by EPO, conformational change of the EPOR/*β*cR heterodimer, phosphorylation of JAK2, and activation of a series of signal transduction proteins that mediate cell protection only require a brief contact with EPO [34]. Consistent with our findings in a study performed on cell cultures of mesenchymal origin, EPOR and *β*cR are barely present in quiescent cells of the cortical region of the kidney. This study by Bohr et al. evaluated the profile of cell surface expression of *β*cR (CD 131) in cells of mesenchymal origin at baseline or under the influence of cell stress induced by lipopolysaccharides (LPS), transforming necrosis factor (TNF-*α*), or an oxidative agent and the effect of a pharmacological-designed derivative of EPO (ARA290) and EPO. Cell stress induced by either LPS, TNF-*α*, or oxidative stress was found to have an impact of at least a twofold increment of CD131-positive cells in cultures, in contrast to cells only exposed to ARA290 or EPO which had no impact in the cell surface migration of CD131 or EPOR from the cytosolic compartment [35].

In our study, the Ad-CKD group displayed EPOR expression and an increased expression of *β*cR; however, no colocalization of receptors can be seen. It is possible that coexpression of EPOR/*β*cR was induced at an earlier time point in the Ad-CKD group (data not shown), when injurious stimuli produced an acute inflammatory response in this study group; further investigations are needed to evaluate this hypothesis. Interestingly, Ad-CKD+rEPO-treated rats showed an increased expression of both parts of the heteroreceptor when compared with the control and Ad-CKD groups and coimmunoprecipitation (Figure 4) and coimmunostaining (Figure 5) of both receptors were evident. The Ad-CKD group treated with rEPO displays increased expression of both receptors individually and coexpression in the merged picture, suggesting that cells can still attempt to activate repair mechanisms as a response to persistent injury, even though some tissue fibrosis have been established at this time in the study. This finding suggests that if sustained tissue injury is capable of stimulating expression of *β*cR, rEPO administration can sustain the coexpression of both parts of the receptor and elicit a protective effect of the tissue, as can be demonstrated by maintaining the expression of AQP1, reducing inflammation and fibrosis (Figures 1–3), and finally improving some functional kidney markers (Table 1). Previous studies show that these effects seem to be mediated by the coexpression of the heterodimer formed by the EPOR and *β*cR receptors and are accomplished by the modulatory effects that rEPO has on inflammation, fibrosis, and apoptosis [33, 34, 36].

Our findings suggest that rEPO can delay or reduce progression of CKD in an animal model of disease and this correlates well with the EPOR/*β*cR heteroreceptor expression. Administration of a weekly dose of rEPO in Ad-CKD rats improved functional parameters of kidney function at 28 days and diminished inflammatory cell migration and deposition of extracellular matrix proteins that result in attenuation of fibrosis. This is a major effect in the renal tissue homeostasis that involves several types of immune cells and is not possible to reach spontaneously [37, 38]. Most studies concerning the effect of rEPO on renal disease are focused on the early stages of damage; however, our study evaluates the effect of rEPO in CKD, and the data suggest that rEPO in a spaced time dosing has a positive impact on GFR, inflammation, and fibrosis.

## Figures and Tables

**Table 1:** Metabolic, hematology, and kidney function parameters.

Values	CTL	Ad-CKD	Ad-CKD+rEPO
Metabolic			
Weight (g)	362.50 ± 9.40	299.00 ± 7.40^ǂ^	322.10 ± 5.40
Food intake (g)	40.00 ± 0.42	23.14 ± 4.46^ǂ^	27.64 ± 0.46^ǂ^
Water intake (mL)	25.58 ± 9.12	61.67 ± 10.14^ǂ^	71.94 ± 8.34^ǂ^
Hematology			
Hemoglobin (g/dL)	16.30 ± 0.14	13.30 ± 0.42^ǂ^	17.80 ± 0.89^∗^
Reticulocytes (%)	3.20 ± 0.40	1.20 ± 0.20^ǂ^	4.00 ± 0.30^∗^
Erythrocytes (×10^6^/*μ*L)	8.50 ± 0.40	6.70 ± 0.10^ǂ^	8.80 ± 0.50^∗^
Hematocrit (%)	52.80 ± 0.30	41.30 ± 1.30^ǂ^	58.20 ± 3.40^∗^
Kidney weight, size, and function			
Mean kidney weight (g)	1.14 ± 0.05	2.80 ± 0.20^ǂ^	1.80 ± 0.11^∗^
Mean kidney size (cm)	1.70 ± 0.09	2.30 ± 0.08^ǂ^	1.90 ± 0.04^∗^
Urinary volume (mL)	16.70 ± 1.90	66.00 ± 7.0^ǂ^	59.80 ± 2.80^ǂ^
Serum creatinine (mg/dL)	0.62 ± 0.04	2.19 ± 0.42^ǂ^	0.93 ± 0.09^∗^
Urinary creatinine (mg/dL)	46.75 ± 8.40	19.00 ± 1.40^ǂ^	19.30 ± 2.20^ǂ^
Urea (mg/dL)	43.50 ± 1.50	196.60 ± 33.40^ǂ^	78.20 ± 5.30^∗^
GFR (mL/min/g)	0.73 ± 0.05	0.24 ± 0.08^ǂ^	0.47 ± 0.08

Values presented as mean ± SD, one-way ANOVA. ǂ represents *P* < 0.05 vs. the control group and ∗ represents *P* < 0.05 vs. the Ad-CKD group vs. the Ad-CKD+rEPO group.

**Table 2:** Semiquantitative scoring for renal injury in experimental groups.

Lesion	CTL	Ad-CKD	Ad-CKD+rEPO
Tubular damage	-	++++	+++
Inflammatory infiltrate	+	++++	+++
Interstitial fibrosis	-	++++	+++

Tubular damage: - = no alteration; + = <25% mild altered tubules; ++ = 25-50% moderate altered tubules; +++ = more than 50% severe altered tubules; ++++ = 75-100% damage area. Inflammatory infiltrate: + = normal; ++ = 10%; +++ = 10-50%; ++++ = more than 50%. Fibrosis: - = no fibrosis; + = mild fibrosis; ++ = 50%; +++ = 50-75%; ++++ = 75-100% fibrosis area.

## Data Availability

The data used to support the findings of this study are included within the article. The data used to support the findings of this study are included within the supplementary information file(s).

## Supplementary Materials

Supplementary 1 Dose selection test and rEPO serum concentration. Rats were administered one of the different dosing schemes of rEPO via s.c: 150 IU/kg of b.w. thrice weekly, 450 IU/kg twice weekly, or 1050 IU/kg once weekly. (a) Red blood cell counts and (b) hemoglobin concentrations were analyzed at 72 h post administration. Both 150 IU/kg and 450 IU/kg doses, three or twice a week, respectively, increased hemoglobin values and red cell counts above the reference ranges. In order to avoid possible thrombotic events, the administration of a weekly dose of 1050 IU/kg was selected. (c) rEPO serum concentrations. rEPO 1050 IU/kg of b.w. via s.c., once weekly, results in peak serum concentrations at 12 h. Detectable serum concentrations are still present 3 days after administration and return to basal levels on the sixth day.

Supplementary 2 Video 1: in the Ad-CKD group, isolated staining of EPOR (red dots) can be seen; however, little *β*cR (green dots) of immunofluorescence can be detected, and no colocalization of both receptors can be identified.

Supplementary 3 Video 2: in the Ad-CKD+rEPO group, EPOR staining is depicted in a regular pattern around tubules (red staining) and colocalization with *β*cR (in green) can be seen as orange dots that can be seen together in tubules.
