# Supplementary figures and images for: Recombinant Erythropoietin Provides Protection against Renal Fibrosis in Adenine-Induced Chronic Kidney Disease
Source: Mediators Inflamm. 2020 Feb 27;2020:8937657. doi: 10.1155/2020/8937657 (PMC7063184; doi:10.1155/2020/8937657)

a)

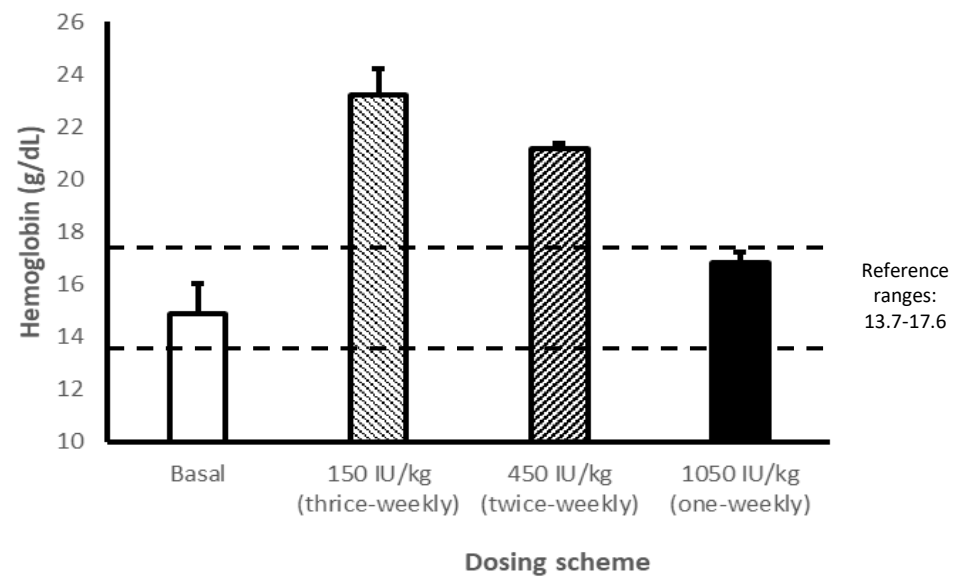

b)

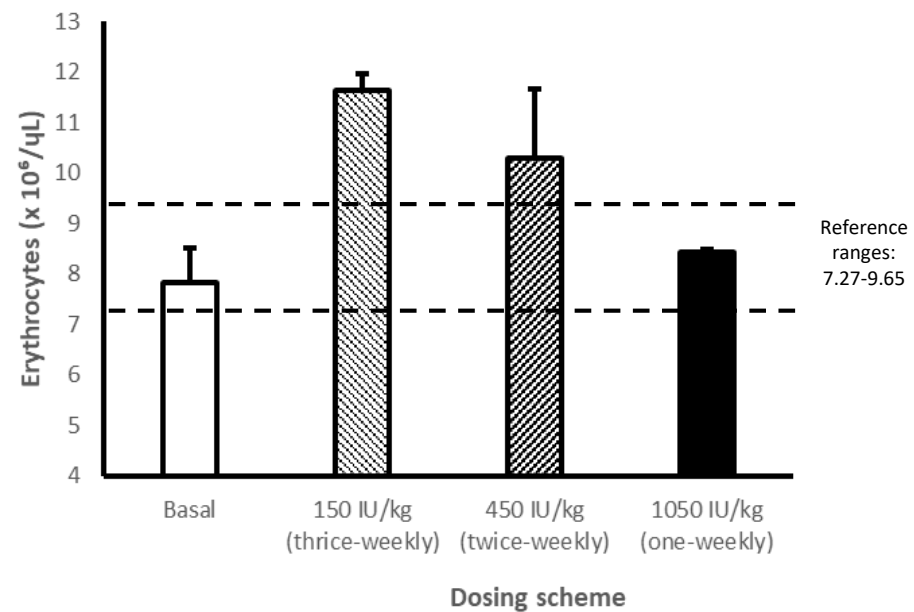

rEPO kinetic (1050 UI/kg via s.c)

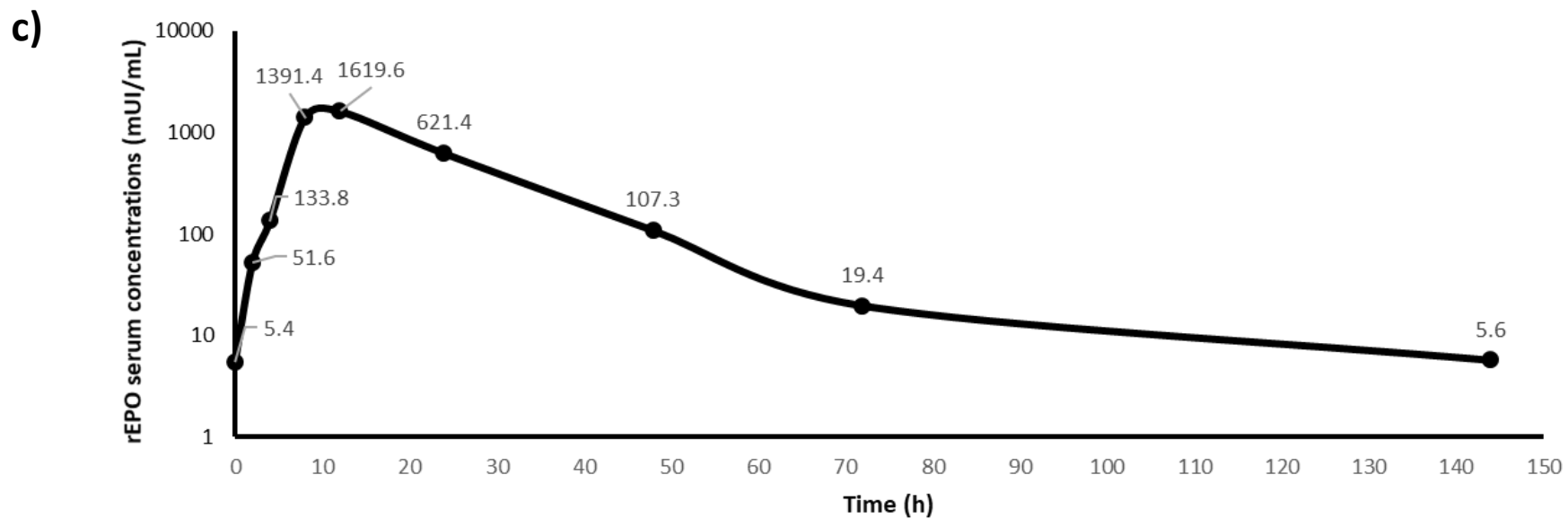

Supplement: Supplementary 1 — Dose selection test and rEPO serum concentration. Rats were administered one of the different dosing schemes of rEPO via s.c: 150 IU/kg of b.w. thrice weekly, 450 IU/kg twice weekly, or 1050 IU/kg once weekly. (a) Red blood cell counts and (b) hemoglobin concentrations were analyzed at 72 h post administration. Both 150 IU/kg and 450 IU/kg doses, three or twice a week, respectively, increased hemoglobin values and red cell counts above the reference ranges. In order to avoid possible thrombotic events, the administration of a weekly dose of 1050 IU/kg was selected. (c) rEPO serum concentrations. rEPO 1050 IU/kg of b.w. via s.c., once weekly, results in peak serum concentrations at 12 h. Detectable serum concentrations are still present 3 days after administration and return to basal levels on the sixth day. [file 8937657.f1.pdf]
